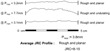# Experimental Characterization of Stress- and Strain-Dependent Stiffness in Grouted Rock Masses

**DOI:** 10.3390/ma11040524

**Published:** 2018-03-29

**Authors:** Ji-Won Kim, Song-Hun Chong, Gye-Chun Cho

**Affiliations:** 1 Department of Civil and Environmental Engineering, Korea Advanced Institute of Science and Technology, 291 Daehak-ro, Yuseong-gu, Daejeon 34141, Korea; g1kim@kaist.ac.kr; 2 Department of Civil Engineering, Sunchon National University, 225 Jungang-ro, Suncheon 57922, Korea; shchong@scnu.ac.kr

**Keywords:** rock grouting, equivalent continuum, shear wave propagation, strain-dependency, modulus degradation

## Abstract

Grouting of fractured rock mass prior to excavation results in grout-filled discontinuities that govern the deformation characteristics of a site. The influence of joint characteristics on the properties of grouted rocks is important in assessing the effects of grouting on jointed rock mass. However, grouting remains a predominantly empirical practice and the effects of grouting on rock joint behavior and material properties have yet to be accurately assessed. Granular materials, including jointed rocks, typically display nonlinear strain-dependent responses that can be characterized by the shear modulus degradation curve. In this study, the effects of grouting on the strain-dependent shear stiffness of jointed rock mass were investigated at the small-strain (below 10^−5^) and mid-strain (10^−5^ to 10^−3^) ranges using the quasi-static resonant column test and rock mass dynamic test devices. The effects of curing time, axial stress, initial joint roughness, and grouted joint thickness were examined. The results show that (1) grouting of rock joints leads to decreased stress sensitivity and increased small-strain shear stiffness for all tested samples; (2) the grouted rock samples display similar modulus degradation characteristics as the applied grout material; (3) the initial joint roughness determines the stress-dependent behaviors and general stiffness range of the jointed and grouted rocks, but the strain-dependent behaviors are dependent on the properties of the grout material; (4) increased grouted joint thickness results in larger contribution of the grout properties in the overall grouted rock mass.

## 1. Introduction

Pre-grouting of fractured rock mass before excavation is essential to improving weak rock zones, such as faults or major joints, to ensure safe construction of underground structures [1,2,3]. Grout injected into anisotropic and discontinuous rock media flows through the fracture network of the host rock, and the grout-filled discontinuities govern the deformation characteristics of the site [4,5,6]. For effective grouting, joint properties, such as the nature of the joint surfaces, their stiffness, frequency, continuity, and interconnection, must be considered [7]. After pre-grouting, the subsequent excavation results in large local non-linear strains within the grouted rock matrix. To determine the accurate shear deformation characteristics of the underground structure, the nonlinear stress- and strain-dependent properties of the grouted rock mass must be included in the safety analysis. However, previous studies on rock grouting focus on the numerical simulation of grout flow in fractures [8,9,10] or field case studies [11,12,13,14] and less emphasis is placed on the actual deformation properties of the site after grouting. Hence, an accurate assessment of the nonlinear stress- and strain-dependent characteristics of grouted rock mass is needed, and the effects of grouting and initial joint properties needs to be investigated.

Elastic wave velocity techniques have been extensively used to evaluate the effects of grouting on the in situ properties of rock masses [11,12,13,14,15,16,17,18]. The presence of joints in the rock media affects elastic wave propagation and attenuation, which is significantly affected by the subjected strain levels, stress state, and joint conditions such as joint roughness or fill materials. For lab experimentation, a jointed rock mass specimen can be modelled using the equivalent continuum model [19]. When the wavelength of the elastic wave travelling through a jointed rock specimen exceeds the length scale of the jointed rock mass, the wave can be considered as propagating in an equivalent continuum. Using this equivalent continuum model, Fratta and Santamarina (2002) [19] developed the quasi-static resonant column test (QRCT), which simulates long wavelength shear wave propagation in cylindrical multi-jointed rock specimens created by stacking multiple rock disks. Cha et al. (2009) [20] expanded the QRCT setup to include a compression wave measuring system. They tested the effects of disk thickness, two-dimensional (2D) joint roughness, joint fill (gouge) material, loading history, and joint cementation. Moreover, Mohd-Nordin et al. (2014) [21] used the same test setup to examine the effects of a three-dimensional (3D) joint roughness profile on wave propagation characteristics, and they correlated that with the joint roughness coefficient. However, previous studies on long wavelength propagation in multi-jointed rocks have been limited to the small-strain domain.

Recent studies have used modified resonant column tests to examine the strain-dependent properties of intact, jointed, or joint-filled rocks [22,23,24]. Chong et al. (2014) [22] developed the rock mass dynamic test (RMDT) device, which can determine the strain-dependent shear modulus and damping ratio of cylindrical multi-jointed rock columns. The device uses the same specimens as the QRCT setup. The researchers conducted tests on a jointed gneiss specimen for different axial stresses and joint fill materials. Perino and Barla (2015) [23] used a fixed-free-type resonant column apparatus (RCA) to test intact and jointed biocalcarenite rock specimens and to examine the effects of smooth and toothed joints. The test setup was validated using 3D DEM numerical modeling. Furthermore, Sebastian and Sitharam (2016) [24] used single-jointed plaster of Paris specimens to investigate the effects of joint orientation and infill material on strain-dependent shear and compression wave propagation. Despite numerous experimental studies on strain-dependent wave propagation in single or multiple rock joints, the strain-dependent characteristics of grouted joints have yet to be theoretically investigated. The strain-dependent properties of grouted soils have been extensively documented for different soil types, cementing agents, and additives using resonant-column or bender-element tests for a wide range of strain levels [25,26,27,28,29]. However, owing to difficulties in simulating grouted joints and testing methodologies, the strain-dependent behavior of grouted rock joints has yet to be properly assessed.

In this study, the effects of grouting and the initial joint conditions on the strain-dependent deformation characteristics of jointed rocks were investigated using the QRCT and RMDT setups. The shear wave velocity in the small-strain range (below 10^−5^) and the strain-dependent shear modulus within the mid-strain range (10^−5^ to 10^–3^) were examined for hollow cylindrical grout, jointed rock, and grouted rock specimens. The strain-dependent shear stiffness of grout and grouted rocks was investigated for different conditions of axial stress, initial joint roughness, and grouted joint thickness. Comparisons between jointed and grouted rocks were made to evaluate the effects of grouting on grouted rock properties.

## 2. Materials

Traditional cement-based grouts are the most widely used form of grout in rock grouting and consist of only water and cement. Grout injected into fractured rock mass permeates through the path of least resistance, and individual cement grains are deposited on the walls of the fracture network to form a coating. To penetrate finer cracks and fissures, highly penetrable grouts are required for rock grouting. This requires a high water-to-cement ratio, often within the range of 100% to 1000%, which results in a very unstable grout mix, severe bleeding, and a long gel time [30]. The use of microcement grout is favorable because it has a smaller particle size compared to ordinary Portland cement. Hence, it results in greater grout strength, better penetration, and less bleeding. In addition to cement and water, setting agents are often added to control the gel time of grouts for field applications.

In the past, silicate-based agents, such as water glass, were widely applied to control setting times of cementitious grouts. However, silicate grouts lose cohesion and degrade easily in areas of high ground water flow, resulting in poor durability and ground water pollution. This factor makes it unsuitable for permanent ground improvement [27]. Currently, the use of silicate-based setting agents has been prohibited in various European countries [31]. An environmentally compatible alternative is calcium aluminate, which naturally occurs in Portland cement in the form of C_2_A and is often used as a quick-setting agent for shotcrete. Calcium aluminates, such as C_12_A_7_, C_11_A_7_, CaA_2_, and C_4_A_3_S, react with water and Ca(OH)_2_ and CaSO_4_ in cement to form a needle-shaped crystalline matrix that contributes to high early strength gains [32]. For the aforementioned calcium aluminate forms, C_12_A_7_ has the fastest hydration reaction and demonstrates hardening within one to three minutes after water contact.

The grout used in this study was a cement milk composed of microcement and water with a water-to-cement ratio of 100%. Amorphous C_12_A_7_ corresponding to 5% of the microcement mass was added as a setting agent and thoroughly mixed with the dry microcement before the addition of water to achieve an even distribution. A hollow cylindrical grout specimen was created using a polyvinyl chloride (PVC) mold to examine the strain-dependent characteristics of the grout material itself. Jointed rock mass was simulated using stacked hollow rock disks following the same equivalent continuum model and methodology outlined in Fratta and Santamarina (2002) [19]. More than nine rock disks were stacked to achieve long wavelength propagation and avoid Brillouin dispersion [20].

The rock disks used in this study were comprised of granite rocks (Geochang granite and Machun granite) commonly found in South Korea. Smooth-surfaced rock disks were created by cutting rock columns using a diamond saw, and central holes were made using a water jet. Rock disks with an initial joint roughness were made by creating brittle fractures in an intact rock column such that the disks had a 3D joint roughness profile and the adjacent disks were interlocked. The average joint roughness coefficient (JRC) [33] of the initial 3D joint roughness profile was JRC = 8–10. Grouting of rock joints was simulated by cementing contiguous rock disks with the microcement grout for a default grouted joint thickness of 1.5 mm. Different grouted joint thicknesses were tested for 2-mm and 4.5-mm thicknesses. The properties of the grout and jointed rock specimens used in this study are shown in Table 1.

## 3. Experimental Study

### 3.1. Experimental Setup

#### 3.1.1. Quasi-Static Resonant Column Test (QRCT)

**Figure 1:**
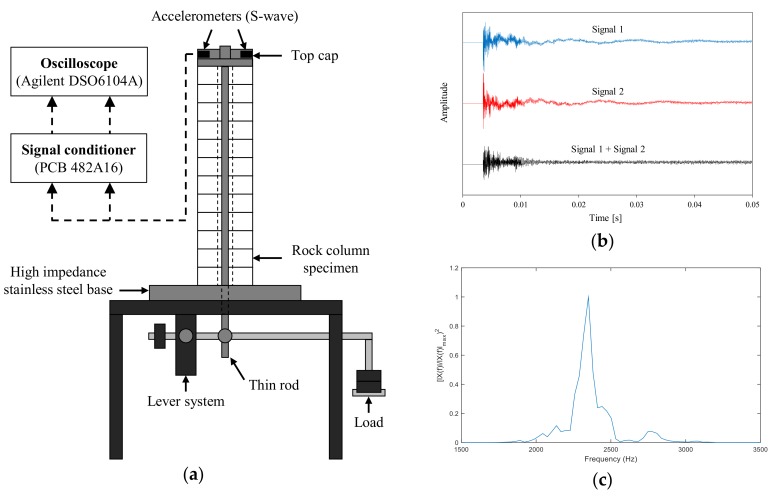
Quasi-static resonant column test (QRCT) apparatus. (**a**) Schematic diagram of the test apparatus; (**b**) Time domain signals obtained from the two accelerometers attached at opposing arms with respect to the axis of rotation. The two signals were added to remove the flexural response; (**c**) Typical frequency domain response curve.

A schematic diagram of the QRCT device setup is shown in Figure 1. The hollow jointed rock specimen was placed on a high-impedance steel base to create a fixed-free boundary condition. An aluminum cap was secured on the top of the specimen, and the axial loading rod was secured to the cap. The axial loading rod ran through the central hole of the specimen and was anchored to the lever system attached below the steel base. Two accelerometers (PCB 352C22) were attached to the top cap at diametrically opposing positions with their axes aligned normal to the radius of the rock column. Torsional excitation was provided by brittle 0.5-mm mechanical pencil lead, which released the column from a static state upon breakage. A signal conditioner (PCB 482A16) was used to amplify the accelerometer signals, and the amplified time-domain signals were added to remove the flexural mode. Further details on the experimental procedures are outlined in Fratta and Santamarina (2002) [19].

The shear wave velocity of the tested specimen could be calculated from its frequency response. The combined time-domain signal obtained from the accelerometers was converted into the frequency domain using fast Fourier transform. The resonant frequency was identified from the first-mode peak resonance. Considering the fixed-free system imposed on the specimen, the wavelength was four times the height of the rock mass. Hence, the shear wave velocity, *V_s_*, could be obtained using the following equation:

(1) $$V_{S}^{QRCT}=\lambda \cdot f_{n}=4L\cdot f_{n}^{QRCT}$$

where *f_n_* is the resonant frequency, *λ* is the wavelength, and *L* is the height of the stacked rock column.

#### 3.1.2. Rock Mass Dynamic Test (RMDT)

**Figure 2:**
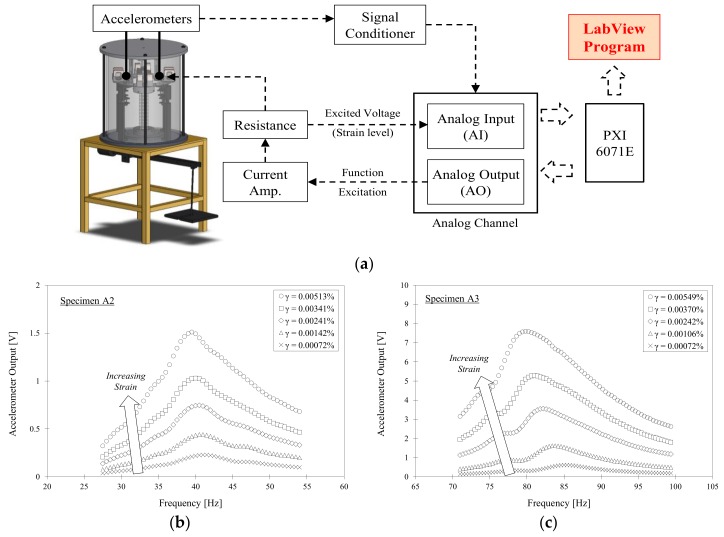
Rock mass dynamic test (RMDT) apparatus. (**a**) Schematic diagram of the test apparatus and data acquisition system (modified from Chong et al., 2014) [22]; (**b**) Frequency response curves for different strain levels (jointed rock specimen); (**c**) Typical frequency domain response curve (grouted rock specimen).

The RMDT device is a modified Stokoe-type resonant column device that has a sufficient torque output (maximum of 30 Nm) to test the rock masses within their nonlinear strain range. The RMDT device consists of four main components; an axial loading system, a torsional driving system, a sensor monitoring system and data acquisition system, as shown in Figure 2 [22]. Axial compression up to 2 MPa can be applied to the rock column using a steel cap-rod-lever system. The necessary fixed-free boundary condition is applied using screw-tightened clippers on the bottom plate and drive cap to secure the specimen in place. The torsional driving system uses a coil-magnet to provide torsional movement. The dynamic response of the specimen was measured using the two accelerometers attached on the top cap. The data was collected using a National Instruments PXI system and analyzed through a LabView program. A predetermined frequency sweep was conducted, and the resonant frequency of the specimen was obtained. The unwanted low-frequency flexural responses are removed by combining the two accelerometer signals as in the QRCT test.

Considering the resonant frequency of the specimen and various input variables of the test apparatus, the shear wave velocity of the specimen could be found using the following equation:

(2) $$\frac{I+I_{c}}{I_{c}}=\frac{2\pi f_{r}l}{V_{S}}\times \tan\left(\frac{2\pi f_{r}l}{V_{S}}\right)$$

where *I* denotes the mass polar moment of inertia of the specimen, *I_c_* is the mass polar moment of inertia of the central rod, *I_d_* represents the mass polar moment of inertia of the drive system secured to the specimen, *l* denotes the specimen length, and *f_r_* represents the specimen resonant frequency obtained from the frequency response curve. The shear modulus (*G*) was then calculated from the shear wave velocity using the following equation:

(3) $$G=\rho \times V_{S}^{2}$$

where *ρ* is the mass density of the specimen. The normalized shear modulus *G*/*G*_max_ relationship is often used to characterize the strain-dependent shear modulus degradation characteristics of various geomaterials. The following hyperbolic model is often adopted for characterizing the strain-dependent shear modulus degradation curve [35]:

(4) $$\frac{G}{G_{\max}}=\frac{1}{1+\left(\frac{\gamma }{\gamma_{ref}}\right)}$$

where *γ_ref_* is the reference strain that corresponds to the strain amplitude when shear modulus reduces to one half of *G*_max_. However, the strain range tested in this study was insufficient to cause large *γ_ref_* values and thus, the normalized modulus degradation curve was plotted to best-fit the experimental data through interpolation.

### 3.2. Experimental Procedure

The effects of grouting, initial joint roughness and joint thickness were tested using the QRCT and RMDT apparatus. The small-strain shear wave velocities were obtained using the QRCT apparatus for 250, 500, 750 and 1000 kPa axial stress. The strain-dependent shear moduli of the same specimens were obtained using the RMDT apparatus for 500 kPa axial stress. The properties of the jointed rock specimen were obtained before grouting. For all grout and grouted rock specimens, the tests were conducted after 7 days of curing. 

The effects of grouting were tested for the Geochang granite specimens before (A2) and after (A3) grouting. The properties of the grout material itself was measured by creating a hollow cylindrical grout specimen (A1). The effects of initial joint roughness were tested for planar-jointed and rough-jointed Geochang granite specimens before (B1 and B2) after grouting (B3 and B4). The effects of grouted joint thickness were tested for the Machun granite specimens with 2 mm (C2) and 4.5 mm (C3) grouted joint thickness and compared with the initial ungrouted condition (C1). The experimental cases for the grout and rock specimens tested in this study are outlined in Table 2.

## 4. Results and Discussion

### 4.1. Effects of Grouting on Jointed Rock Mass

The shear wave velocities of the grout and grouted rock specimens with curing time were initially measured for 7, 14 and 28 days of curing using the quasi-static resonant column test. The grout specimen displayed a gradual increase in shear wave velocity with curing time and the shear wave velocity reached 87.5% of its 28-day cured wave velocity within the first 7 days of curing. Similar observations were made by Kim et al. (2008) [36] where a Joomunjin sand and Portland cement mixture (water-to-cement ratio = 170%) displayed nearly constant elastic wave velocities after 10 days. The grouted rock specimens also displayed similar early stiffness developments, reaching 94.5% of its 28-day cured wave velocity within the first 7 days of curing. The grouted rock specimens are less affected by the curing time of the grout as it only occupies the joints of the entire grouted rock mass. As the majority of the grout wave velocity was reached within 7 days of curing, the results from the 7-day tests were analyzed in this study.

**Figure 3:**
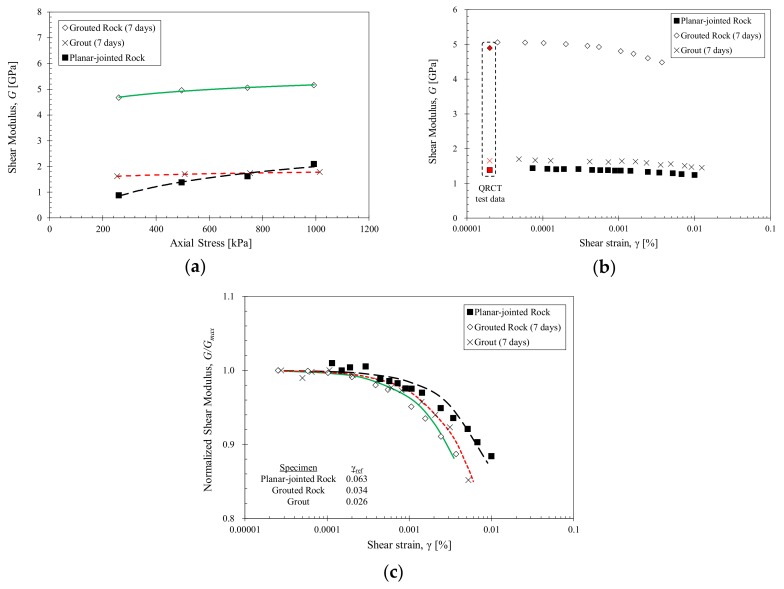
Stress- and strain-dependent properties of the planar-jointed rock, grouted rock (7 days of curing), and grout specimens (7 days of curing). (**a**) Stress-dependent small-strain shear modulus for 250, 500, 750 and 1000 kPa axial stress; (**b**) Strain-dependent shear modulus at 500 kPa axial stress. The small-strain shear modulus shown in red is obtained from the small-strain shear wave velocity; (**c**) Normalized shear modulus degradation curve. The data is fitted using the hyperbolic model.

The QRCT and RMDT test results of the intact grout specimen (A1) and the planar-jointed rock specimen before (A2) and after grouting (A3) with 7 days of curing are shown in Figure 3. The small-strain shear wave velocities displayed in Figure 3a show increased shear wave velocities with increased axial stress for all tested specimens, as increased confinement results in improved wave propagation. With grouting, the shear wave velocity of specimen A3 displayed a significant increase of up to 2.34 times from the initial ungrouted condition. Grouting also resulted in a lower stress dependency compared to the initial condition. The differential between the small-strain wave velocities measured at 250 kPa and 1000 kPa axial stress were 316 m/s for specimen A2, 33 m/s for specimen A3 and 49 m/s for specimen A1. The presence of discontinuous joints in specimen A2 resulted in attenuation and energy losses at the rock-joint interface which contributed to the total loss in the medium and enabled more compressibility with increased stress. As the grout material filled the discontinuous joints, the joint filling and adhesive effects of the grout bound the rock disks into a single grouted rock column. This resulted in increased shear stiffness and decreased stress sensitivity. The similar stress-dependent changes between specimens A2 and A3 imply that the stress-dependent characteristics of grouted planar joints were governed by the characteristics of the grout material.

The strain-dependent shear modulus presented in Figure 3b shows a decrease in shear modulus with increased shear strain levels for all tested specimens. The small-strain shear modulus from the QRCT tests show good correlation with the RMDT results. The shear modulus of specimen A3 is much larger than that of specimens A1 and A2 for all tested strain levels. This shows that grouting resulted in cementation and joint filling, which provided increased shear stiffness and adhesion between rock disks. The normalized shear modulus degradation curves depicted in Figure 3c show that specimen A2 displays a unique curvature characteristic that greatly differs from that of the grout-based specimens A1 and A3. Hardin and Drnevich (1972) [35] noted that the shape of the stress-strain curve is largely dependent on the type of soil material. In the case of jointed rocks, the existence and nonexistence of discontinuities can be accounted for this discrepancy between ungrouted and grouted specimens. Both specimens A1 and A3 display similar degradation characteristics, which indicates that the strain-dependent characteristics of grouted rocks are now governed by the properties of the grout material. A shift has occurred from joint stiffness-based shear characteristics to grout-based shear characteristics.

### 4.2. Effects of Initial Joint Roughness on Grouted Rock Mass

**Figure 4:**
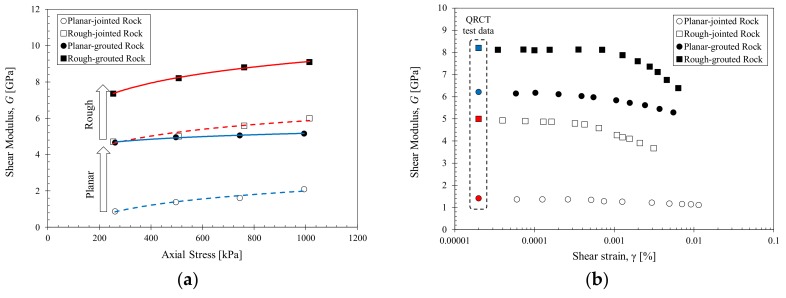

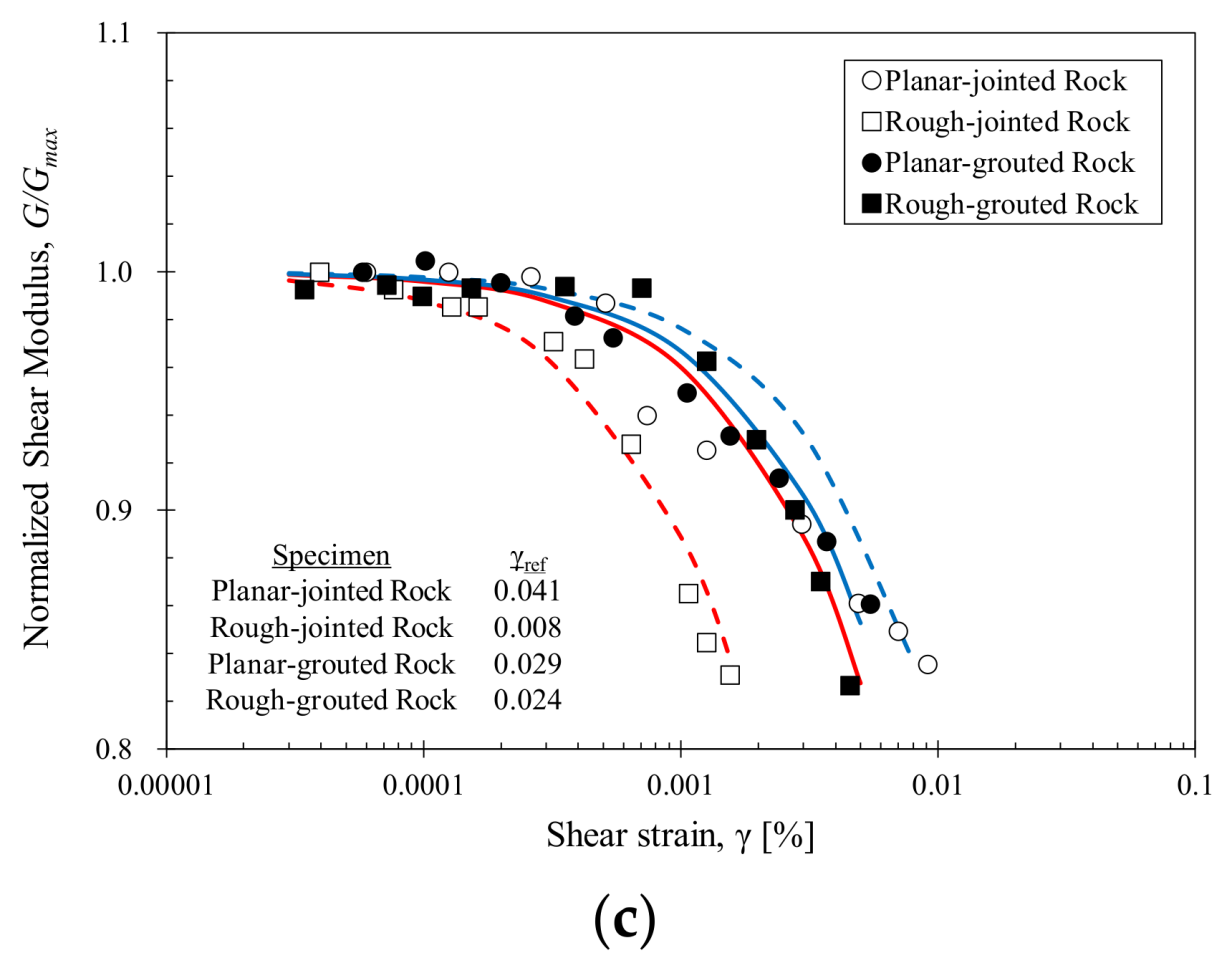
Stress- and strain-dependent properties of the planar-jointed rock and rough-jointed rock before and after grouting and with 7 days of curing. (**a**) Stress-dependent small-strain shear modulus for 250, 500, 750 and 1000 kPa axial stress; (**b**) Strain-dependent shear modulus at 500 kPa axial stress. The small-strain shear modulus shown in red and blue are obtained from the small-strain shear wave velocity; (**c**) Normalized shear modulus degradation curve. The data is fitted using the hyperbolic model.

The QRCT and RMDT test results for the planar-jointed rock and rough-jointed rock specimens before and after grouting are shown in Figure 4. The rough-jointed specimen B2 displays larger small-strain shear wave velocity and stress-dependency compared to the planar-jointed specimen B1, which is consistent with results from previous studies on the 3D joint roughness profile [37]. The rough joint surface allows interlocking and increases the contact area between contiguous joints, resulting in increased shear wave propagation. With grouting, both specimens B1 and B2 display increases in shear wave velocity as the joint filling and cohesion effects of grout bind the rock disks into a single grouted rock column. The stress dependency of the grouted planar-jointed rock decreases with grouting as the grout provides resistance against loading through joint filling. 

However, as shown in Figure 4a, the stress dependency of specimen B4 displays similar stress-dependent trends with its initial ungrouted condition. While the differential between the small-strain wave velocities measured at 250 kPa and 1000 kPa axial stress are 307 m/s and 97 m/s for specimens B1 and B3, the differential is 170 m/s and 187 m/s for specimens B2 and B4. This small stress dependency is related to the characteristics of 3D joint roughness profiles. For grouted rough joints, the irregular joint roughness causes a variation in the grout-fill within joints. Planar-grouted joints have a clear distinction between the rock disks and grouted joints. On the other hand, rough-grouted joints do not have a clear distinction between the grout material and protruding rock elements caused by initial joint roughness. This mixture of rock and grout in rough-grouted joints contribute differently to the overall stiffness of the grout-filled joints. Since the intact rock disk has a higher stiffness than the grout material, the presence of rock elements indicate that the shear stiffness of grouted rough joints will be governed dominantly by the shear stiffness of the rough joints, rather than the grout material. 

The strain-dependent shear modulus presented in Figure 4b shows a decrease in shear modulus with increased shear strain levels for all tested specimens. For all measured strain levels, specimen B2 displayed a higher shear modulus compared to specimen B1 as the interlocking between rough joints increased the initial joint shear stiffness. With grouting, both the planar-jointed and rough-jointed specimens displayed increased shear stiffness from their initial values.

The increase in shear modulus was similar for both specimens regardless of their initial joint roughness; specimen B3 increased by 3.62 GPa from its initial B1 state and specimen B4 increased by 3.18 GPa from its initial B2 state. The normalized shear modulus degradation curves of the two initial jointed rock specimens displayed markedly different trends, wherein the rough-jointed rock displayed a much shorter linear range (*γ_ref_* = 0.008%) compared to the planar-jointed rock (*γ_ref_* = 0.041%). With grouting, the two grouted rock specimens demonstrated comparable strain-dependent characteristics, and their reference strains formed at similar values between the two initial conditions. This similar increase in shear modulus and similar strain-dependent stiffness degradation indicated that the shear stiffness of the grouted rocks is governed by the properties of the grout material. The initial joint roughness is reflected in the stress-dependent stiffness and general stiffness range of the specimen, with rough interlocked joints displaying higher shear stiffness and less stress-dependent changes for both ungrouted and grouted conditions compared to planar joints.

### 4.3. Effects of Grouted Joint Thickness on Grouted Rock Mass

**Figure 5:**
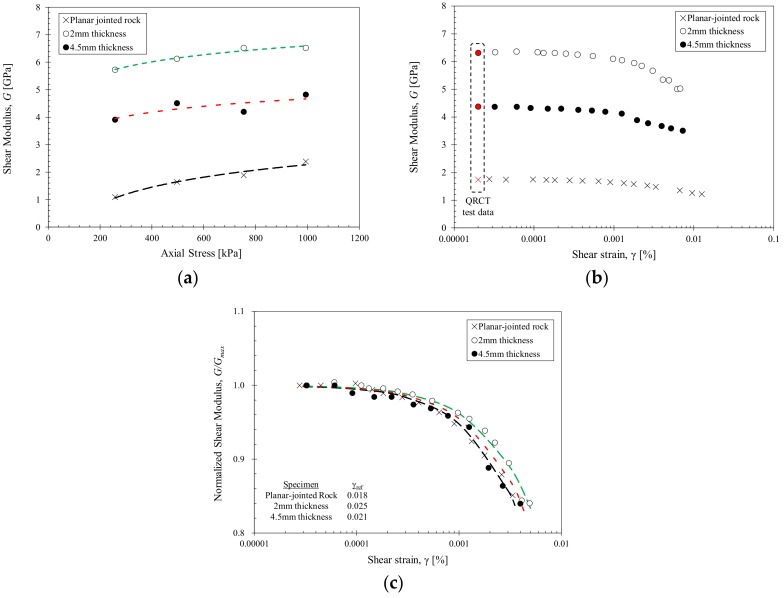
Stress- and strain-dependent properties of the planar-jointed rock with a grouted joint thickness of 2 mm and 4.5 mm after 7 days of curing. The properties of the planar-jointed rock are shown as a reference. (**a**) Stress-dependent small-strain shear modulus for 250, 500, 750 and 1000 kPa axial stress; (**b**) Strain-dependent shear modulus at 500 kPa axial stress. The small-strain shear modulus shown in red is obtained from the small-strain shear wave velocity; (**c**) Normalized shear modulus degradation curve. The data is fitted using the hyperbolic model.

Figure 5 presents the QRCT and RMDT test results for the planar-jointed rock specimens with 2 mm and 4.5 mm grouted joint thickness. For all tested stress levels, specimen C2 displays a higher shear wave velocity and shear modulus compared to specimen C3. This is attributed to the adhesion and joint filling effects of the grouted joints. A grouted joint is comprised of a joint filling zone and shear contribution zone. The rock-grout interface contributes to the shear behavior of the grouted rock mass (shear contribution zone); the other parts of the grouted joint contributed to the joint filling effect (joint filling zone). The shear contribution zone is limited to the rock-grout interface and is independent of the grouted joint thickness. This is indicated by the similar stress- and strain-dependent characteristics between specimens C2 and C3. The differential between the small-strain wave velocities measured at 250 kPa and 1000 kPa axial stress are 148 m/s and 130 m/s respectively for specimens C2 and C3. The normalized shear modulus degradation curves also display similar characteristics, with the reference strains of 0.025 and 0.021 respectively for specimens C2 and C3.

The grouted joint thickness has a major effect on the general stiffness range of the grouted rock specimens. Thicker grouted joints and larger joint filling zones resulted in a larger contribution of the grout material properties in the grouted rock specimen, which had comparatively lower shear stiffness compared to the intact rock material. Increasing the grouted joint thickness implies larger contribution of the grout properties, resulting in decreased shear stiffness of the overall grouted rock specimen. The largest shear modulus increase would be observed when the joint filling zone is minimized with the maximum shear contribution zone.

If the grout material used in this study had a higher shear stiffness compared to the rock disks, the grout would have a more dominant effect on the properties of the grouted rock. More sensitive changes in the shear stiffness would be observed with grouting, as well as with increasing grouted joint thickness. In addition, more governance of the grout properties in terms of the overall stiffness of the grouted rock specimen would be observed. Further studies considering grout materials of varying shear stiffness are needed to verify this idea.

## 5. Analysis

**Figure 6:**
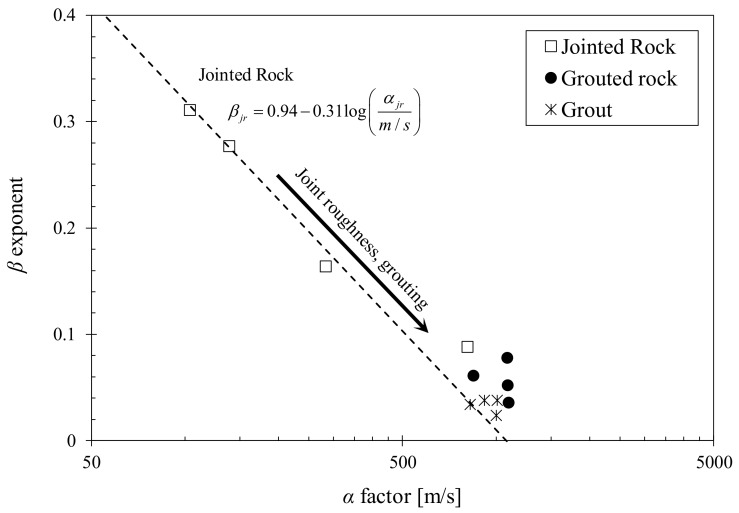
*α* and *β* parameters of the Hertzian power function for all tested grout and rock specimens (The trend line for jointed rock specimens is adopted from Cha et al. (2014) [39]).

Previous studies on shear wave propagation in jointed rocks in the small strain domain showed that the shear wave velocity can be expressed using the Hertzian power function. The following equation can be used to fit the long wavelength wave velocity data versus the axial stress [20,38]:

(5) $$V_{S}=\alpha {\left(\frac{\sigma_{n}}{1kPa}\right)}^{\beta }$$

where *α* is the wave velocity at 1 kPa axial stress and *β* is the stress sensitivity. The *α* values are plotted against the *β* exponents for the tested jointed and grouted rocks in Figure 6. For comparison, the fitted equations for jointed rock data obtained from previous laboratory studies are superimposed. The planar-jointed rock specimens tested in this study showed a good fit with the jointed rock trends from previous studies. The interlocked rough-jointed rock however, displayed similar low stress sensitivity and high wave velocity characteristics to grouted rocks as the interlocking effect between rock disks improved the shear stiffness of the specimen. Although only 2D joint roughness profiles are considered in the study by Cha et al. (2014) [39], the results from specimens with 3D joint roughness profiles tested in this study fit the suggested equation well. Mohd-Nordin al. (2014) [31] noted that the shear wave velocity ratio between rough jointed and intact rock columns decreased with significantly for axial stresses larger than 100 kPa. As the shear wave velocities in Cha et al. (2009) [37] were measured up to approximately 700 kPa, the fitted equation can hold for specimens with 3D joint roughness profiles.

Grouted rock specimens do not follow the same fitted line as jointed rocks and are distributed in a unique range spanning *α* values from 845 to 1241 and *β* exponents from 0.016 to 0.078. This is attributed to the filling and cementation of joints through grouting, which leads to increased stress resistivity and improved elastic wave propagation. The range of *α* and *β* values is similar to that of intact grout specimens, with *α* values spanning from 827 to 1008 and *β* exponents from 0.024 to 0.038. This similarity indicates that the properties of the grout material greatly influences the stress- dependent wave propagation and stiffness of grouted rocks. The range of *α* and *β* values for grouts and grouted rocks would vary depending on the stiffness of the grout material. Additional experimental and theoretical studies using different grout materials and different stiffness ratios between the grout and intact rock are required for more comprehensive analysis.

## 6. Conclusions

In this study, an experimental investigation was conducted on the stress- and strain-dependent stiffness of grout, jointed rock and grouted rock specimens for different axial stress, initial joint roughness, and grouted joint thickness conditions. The shear wave velocity in the small-strain range (below 10^−5^) and the strain-dependent shear modulus within the mid-strain range (10^−5^ to 10^–3^) were tested for axial stresses up to 1000 kPa. The main findings of this study are as follows:

(1) The presence of discontinuous joints in the rock specimens resulted in attenuation and energy losses at the rock-joint interface, which contributed to the total loss in the medium and enabled more stress-dependent compressibility. As the grout material filled the discontinuous joints, the joint filling and adhesive effects of the grout bound the rock disks into a single grouted rock column. This resulted in increased shear stiffness and decreased stress sensitivity.
(2) Grouting of rock joints led to improved shear stiffness and decreased stress-dependency for all tested stress and strain levels. Both the intact grout and planar-grouted rock specimens (A1 and A3) displayed similar degradation characteristics with increasing shear strain, which indicated that the strain-dependent characteristics of grouted rocks are governed by the properties of the grout material.
(3) Rough interlocked joints (B2) have higher shear stiffness compared to planar joints (B1) as the rough joint surface allows interlocking and increased contact area between contiguous joints. Rough-jointed joints also displayed a much shorter linear range for strain-dependent modulus degradation.
(4) Grouted rough and planar joints (B3 and B4) displayed similar increases in shear modulus and strain-dependent characteristics with grouting. This indicated that the strain-dependent shear stiffness of the grouted rocks is predominantly governed by the properties of the grout material. The stress-dependent stiffness is influenced by the initial joint roughness, where the rough interlocked joints displayed a unique shear stiffness for both ungrouted and grouted conditions (B2 and B4).
(5) The rock-grout interface contributes to the shear behavior of the grouted rock mass (shear contribution zone) while the other parts of the grouted joint contributed to the joint filling effect (joint filling zone). The shear contribution zone is limited to the rock-grout interface and is independent of the grouted joint thickness. Increased grouted joint thickness implies larger contribution of the grout properties, resulting in decreased shear stiffness of the overall grouted rock specimen. The largest shear modulus increase would be observed when the joint filling zone is minimized with the maximum shear contribution zone.
(6) The range of *α* (wave velocity at 1 kPa axial stress) and *β* (stress sensitivity) values of jointed rocks follow the general trend line outlined in Cha et al. (2014) [39]. Grouts and grouted rocks form a similar unique distribution characterized by low stress sensitivity and high wave velocities. This similarity indicates that the properties of the grout material greatly influences the stress- dependent wave propagation and stiffness of grouted rocks. The range of *α* and *β* values for grouts and grouted rocks vary depending on the stiffness of the grout material.

Further studies on different grout materials, different stiffness ratios between the grout and intact rock and initial joint roughness are required to provide more comprehensive analysis on the effects of grouting in jointed rock masses. The results of this study can be applied to seismic design or safety analysis of underground construction works.

## Figures and Tables

**Table 1:** Specification of rock specimens used in this study.

Configuration	Geochang Granite				Machun Granite		Grout ^1^
Joint Condition	Intact	Planar	Planar	Rough	Planar	Planar	Intact
Average grouted thickness (mm)	-	1.5	1.5	1.5	2.0	4.5	-
Outer diameter (mm)	62.4	62.4	51.5	51.7	63.0	63.0	67.0
Inner diameter (mm)	25.0	25.0	15.0	15.0	24.0	24.0	25.0
Length (mm)	295.0	311.0	301.5	309.0	268.5	291.0	290.0
Density (kg/m^3^)	2789	2589	2600	2628	2797	2714	1580
Number of disks in column ^2^	-	12	12	12	10	10	-
Intact rod wave velocity (m/s) ^3^	3893				4703		858
Intact P-wave velocity (m/s) ^4^	4082				4918		876
Intact S-wave velocity (m/s) ^5^	2522				3053		570
Poisson’s ratio *ν* ^6^	0.19				0.19		0.13

^1^ The grout specimen was tested after 7 days of curing in water; ^2^ The Machun granite specimen had ten disks in the column owing to the specimen height limitations of the test apparatus; ^3^ Intact rod wave velocities obtained from free-free resonant column tests conducted on intact rock specimens using the first mode resonance frequency [34]; ^4^ Intact P-wave velocities obtained from point-source travel time tests conducted on intact rock specimens using the travel time of wave propagation [34]; ^5^ Intact S-wave velocities were estimated using the following theoretical relationship: $\nu =\frac{V_{P}^{2}\;-\;2V_{S}^{2}}{2\left(V_{P}^{2}\;-\;V_{S}^{2}\right)}$; ^6^ Poisson’s ratio was indirectly calculated from the rod wave velocity and P-wave velocity using the following equation: $\frac{V_{rod}^{2}}{V_{P}^{2}}=\frac{\left(1\;+\;\nu \right)\left(1\;-\;2\nu \right)}{\left(1\;-\;\nu \right)}$.

**Table 2:** Experimental cases for grout and rock specimens tested in this study.

Configuration		Specimen Description	Specimen Image	Joint Roughness Condition	Average Joint Thickness
Effects of grouting	A1	Grout	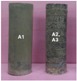	-	-
	A2	Planar-jointed rock		Planar	Negligible (non-filled)
	A3	Grouted planar-jointed rock		Planar	1.5 mm
Effects of initial joint roughness	B1	Planar-jointed rock	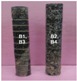	Planar	Negligible (non-filled)
	B2	Rough-jointed rock		Rough	Negligible (non-filled)
	B3	Grouted planar-jointed rock		Planar	1.5 mm
	B4	Grouted rough-jointed rock		Rough	1.5 mm
Effects of joint thickness	C1	Planar-jointed rock	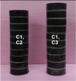	Planar	Negligible (non-filled)
	C2	Grouted planar-jointed rock		Planar	2.0 mm
	C3	Grouted planar-jointed rock		Planar	4.5 mm
Note: Initial joint roughness profile was formed by intentionally fracturing an intact rock column. The average JRC was 8–10 according to the JRC profile characteristics outlined by Barton and Choubey (1977) [33].			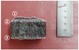